# Botanical Compounds: Effects on Major Eye Diseases

**DOI:** 10.1155/2013/549174

**Published:** 2013-06-17

**Authors:** Tuan-Phat Huynh, Shivani N. Mann, Nawajes A. Mandal

**Affiliations:** ^1^Department of Ophthalmology, The University of Oklahoma Health Sciences Center, Oklahoma City, OK 73104, USA; ^2^Dean A. McGee Eye Institute, 608 Stanton L Young Boulevard, Oklahoma City, OK 73104, USA; ^3^College of Public Health, The University of Oklahoma Health Sciences Center, Oklahoma City, OK 73104, USA; ^4^Department of Physiology, The University of Oklahoma Health Sciences Center, Oklahoma City, OK 73104, USA; ^5^The Oklahoma Center for Neuroscience, The University of Oklahoma Health Sciences Center, Oklahoma City, OK 73104, USA

## Abstract

Botanical compounds have been widely used throughout history as cures for various diseases and ailments. Many of these compounds exhibit strong antioxidative, anti-inflammatory, and antiapoptotic properties. These are also common damaging mechanisms apparent in several ocular diseases, including age-related macular degeneration (AMD), glaucoma, diabetic retinopathy, cataract, and retinitis pigmentosa. In recent years, there have been many epidemiological and clinical studies that have demonstrated the beneficial effects of plant-derived compounds, such as curcumin, lutein and zeaxanthin, danshen, ginseng, and many more, on these ocular pathologies. Studies in cell cultures and animal models showed promising results for their uses in eye diseases. While there are many apparent significant correlations, further investigation is needed to uncover the mechanistic pathways of these botanical compounds in order to reach widespread pharmaceutical use and provide noninvasive alternatives for prevention and treatments of the major eye diseases.

## 1. Introduction

Botanical compounds have a long history of medicinal use. The earliest record of plants in medicine was found on clay tablets from Mesopotamia around 2600 B.C. This depiction showed the use of oils from *Cupressus sempervirens*, also known as cypress, in the treatment of coughing, colds, and inflammation [1]. Today, many natural compounds from plants are active ingredients in the fabrication of modern drugs. For example, the active ingredient of aspirin is acetylsalicylic acid, which is derived from a compound called salicin that is isolated from the bark of *Salix alba L,* a species of willow tree. Similarly, analyses of *Papaver somniferum L.*, commonly called opium poppy, lead to the discovery of numerous alkaloids, including morphine [2]. Currently, 74% of modern drugs directly used in traditional medicine have their origins in natural compounds. Numerous botanical compounds contain active ingredients or produce secondary metabolites that have beneficial properties, including anti-inflammation, antioxidation, protection against apoptosis, and restoration of the body's homeostasis. As the pathologic mechanisms of major blinding diseases, such as age-related macular degeneration (AMD), diabetic retinopathy (DR), cataracts, and glaucoma, often involve inflammation- and oxidative stress-mediated cell death, evidences are accumulating on the potential benefits of botanical compounds in diets to improve or prevent these vision threatening eye diseases [3]. It is estimated that 35 million Americans combined suffer from glaucoma, cataracts, and AMD, the leading causes of blindness in the country [4, 5]. Despite this high prevalence, there are few or no treatments currently available for diseases such as AMD and glaucoma. Preventive intervention may, therefore, be the most effective course of action against these age-related ocular diseases. 

**Figure 1:**
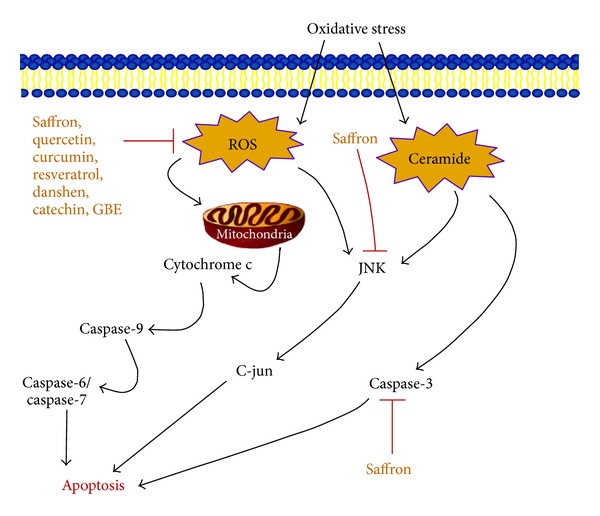
Oxidative stress pathway and botanicals. A schematic representation of cellular oxidative stress pathway and the effects of the botanical compounds discussed in this review that prevent the formation of reactive oxygen species (ROS) and protect the cell from apoptosis. Saffron particularly affects the JNK pathway and the production of caspase-3 from ceramide, which also lead to apoptosis.

**Figure 2:**
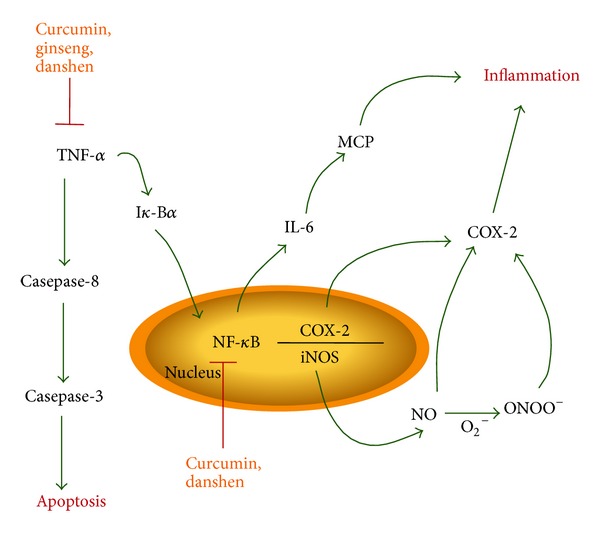
Effects of selected botanical compounds on TNF-*α* and NF-*κ*B pathways. TNF-*α* and NF-*κ*B pathways are the major pathways of cellular stress and inflammation. Botanical compound curcumin and danshen inhibit the activation of TNF-*α* and NF-*κ*B. Ginseng also interferes with the activation TNF-*α* and protects the cell from apoptosis.

According to the World Health Organization, traditional medicines remain the principal source of health care for 80% of the world's population [6], and there is an increased interest in the Western world for functional foods, or “nutraceuticals.” Nutraceuticals are not defined as food but rather as derived products from botanicals and other natural products in the forms of pills, powders, and other types of dietary supplements, which contain active ingredients that have shown potential benefits for human health. The ingredients of botanical functional food and nutraceuticals are classified upon their biochemical properties and benefits, such as antioxidants, anticarcinogens, inflammation-inhibitors, blood pressure reducing agents, or antidiabetics [3]. Recent research studies have identified molecular mechanism of action of many of the botanical compounds, which are in use as nutraceuticals or supplements as summarized in Figures 1 and 2. However, the detail mechanisms of most plant-derived active ingredients are still under investigation, and few research studies have been performed on the efficacy of botanical compounds' effects on human diseases, especially for eye diseases. In this paper, we reviewed the evidence that certain botanical ingredients, including curcumin, lutein, zeaxanthin, saffron, catechin, *Ginkgo biloba* extract, ginseng, resveratrol, danshen, and quercetin, may be used as dietary supplements to have therapeutic benefits for many common ocular diseases. 

## 2. Major Eye Diseases Share Common Mechanistic Pathways

AMD, glaucoma, cataract, and other retinal diseases, including diabetic retinopathy (DR) and retinitis pigmentosa (RP), are the major causes of blindness around the world [4, 5, 7]. An epidemiologic survey conducted by the Eye Diseases Prevalence Research Group indicated that by 2020 an estimated 30.1 million Americans will suffer from cataract [7]. Moreover, it was predicted that during that same year, 2.95 million and 2.2 million Americans will be diagnosed with AMD and glaucoma, respectively, and an estimated 4.1 million Americans aged 40 years and older will be suffering from diabetic retinopathy [4, 5, 8]. Interestingly, all these diseases are associated with aging, and their etiology or pathophysiologies share some common mechanistic pathways. These pathways include oxidative stress, inflammation, and apoptotic factors, which provide insight for potentially targetable areas. Indeed, in many cases of eye diseases, oxidative stress due to reactive oxygen or nitrogen species and lipid peroxidation lead to ocular cell death. In addition, many pathogenic pathways include inflammatory factors such as the tumor necrosis *α* (TNF-*α*) and nuclear factor-kappa B (NF-*κ*B). Interestingly, these pathways often intersect with the mechanism of action of many botanical compounds (Figures 1 and 2). Oxidative stress induces the formations of reactive oxygen species, which interact with the mitochondria and activates the JNK pathway leading to apoptosis. Since AMD, DR, RP, and glaucoma all have a significant impact on populations worldwide, this review will focus on these pathologies and the potential benefits of botanical compounds in their prevention and treatment.

### 2.1. Age-Related Macular Degeneration (AMD)

AMD is a chronic retinal disease, commonly present among populations of age 50 or older, resulting in loss of central vision due to degeneration of photoreceptor and RPE cells in the macula, which are essential in providing sharp and clear vision. Many epidemiologic surveys describe AMD as the major cause of blindness in elderly populations worldwide; risk factors include smoking, race, and family history. The major mechanistic pathways of the pathology include oxidative stress and inflammation [9–11]. The results from the age-related eye disease study (AREDS) of the National Eye Institute suggested that dietary supplements with antioxidants and zinc may decrease the risk of developing advanced AMD and could significantly prevent vision loss [12, 13]. This study opens the horizon for promising natural compounds in the prevention of AMD. Many plants' active ingredients have already been investigated for their antioxidant and anti-inflammatory properties, which may be critical in the treatment and prevention of AMD and other ocular diseases. 

### 2.2. Glaucoma

Glaucoma is described as a group of eye conditions leading to the interruption of visual information from the eye to the brain [14]. In most cases of glaucoma, an increased pressure in the eye, commonly known as intraocular pressure (IOP), causes damage to the optic nerve via retinal ganglion cell (RGC) apoptosis [15]. The major treatment procedure for glaucoma consists of lowering the IOP through eye drops, oral drugs, and even sometimes surgery [16]. Although IOP is one of the main factors in glaucoma, many cases progress despite the lowering of eye pressure to standard levels. In those cases, it is necessary to find new and innovative ways to prevent or limit the damages other than lowering the IOP. Since apoptosis plays a significant role in glaucoma, investigations of compounds described as neuroprotectants may lead to promising results. Numerous botanical compounds possess such neuroprotective properties, which may be effective in the prevention and treatment of glaucoma.

### 2.3. Diabetic Retinopathy (DR) and Retinitis Pigmentosa (RP)

Diabetic Retinopathy occurs in individuals suffering from both type 1 and type 2 diabetes. Along with AMD and glaucoma, DR is a leading cause of blindness worldwide, and it is estimated that 140 million individuals are affected with DR [17]. The pathology is triggered by changes in the retinal blood vessels. Blood vessels may swell or leak and growth of new abnormal vessels may be detected on the retinal surface. There are four stages in the DR pathology: mild nonproliferative retinopathy, moderate nonproliferative retinopathy, severe nonproliferative retinopathy, and proliferative retinopathy. There are no treatments required for the first three stages; however, in order to prevent progression of the disease in these stages, patients are required to control their blood sugar, blood pressure, and blood cholesterol [18]. In cases of proliferative retinopathy, laser surgery is needed to control leaking fluids. The laser treatment helps shrink the abnormal blood vessels, but in cases of severe bleeding a vitrectomy is required to remove the blood from the center of the eye [17, 18]. 

Retinitis pigmentosa (RP) is another retinal disease in which retinal rod and cone cells are affected, leading to decreased vision and, in severe cases, blindness. RP's main risk factor is genetic predisposition, and it is a less common disease, affecting only 1 in 4000 people in the United States [19, 20]. Although it is not as prevalent as other ocular diseases, RP is a significant problem because there are no current treatments. However, previous studies in mice suggested that high doses of antioxidants, such as vitamin A palmitate, may slow the disease [20]. For both RP and DR, many plant extracts may have significant effects on treatment and prevention. However, the lack of clinical trials leaves many uncertainties on the possible benefits of such supplements. Current studies suggest interesting links between dietary supplements and positive effects on subjects with RP and similar retinal diseases. 

### 2.4. Cataract

Cataract is an age-related eye disease that usually occurs starting at age 60. This pathology affects the lens due to the breakdown of proteins, leading to the clouding of the lens. Because the lens is necessary to focus on close or far away objects, damage to it leads to blurry vision with decreased color and shape sensitivity. Several factors, such as smoking, diabetes, eye injury, exposure to ultraviolet (UV) light, and family history, are known to favor cataract formation [21]. Patients with cataract can easily be treated by having surgery, which will remove the opaque lens and replace it with an artificial intraocular lens. While the surgery successfully restores sight in most patients, many countries do not have access to such eye care, and it is estimated that 51% of world blindness is caused by cataract [22]. Fortunately, recent studies have shown that consumption of botanical compounds containing strong antioxidants may prevent the degradation of proteins in the eye and minimize the effects of cataract. 

## 3. Botanical Compounds and Their Effects on Eye Diseases

### 3.1. Curcumin

Curcumin, also known as *Curcuma longa*, is a spice widely prevalent in the South Asian diet. It is extracted from the turmeric plant and has a long history of use against inflammatory diseases. [23]. Curcumin is a lipophilic polyphenol and is insoluble in water, but it can remain stable in acidic pH environments like the human stomach [24, 25]. The mechanisms of curcumin involve interaction with many molecular targets for inflammation. For example, it regulates inflammatory processes by controlling the activity of cyclooxygenase-2 (COX-2), lipoxygenase, and nitric oxide synthase (iNOS) enzymes (Figure 2). Curcumin also inhibits the production of the inflammatory cytokine TNF-*α* as well as interleukins, monocyte chemoattractant protein (MCP), and migration inhibitory protein (Figure 2) [26, 27]. Since curcumin is an anti-inflammatory and antioxidative agent, we analyzed its effect on light-induced retinal degeneration (LIRD) in rat models and on retina-derived cell lines. We observed retinal neuroprotection in rats supplemented with 0.2% curcumin in their diet for two weeks [28]. Curcumin protected the retina from LIRD through inhibition of NF-*κ*B activation and downregulation of inflammatory genes. Experiments on pretreatment of retina-derived cell lines, 661W and ARPE-19, with curcumin lead to protection of the cells from hydrogen peroxide (H_2_O_2_) induced cell death by activating cellular protective enzymes, such as HO-1 and thioredoxin [28]. Further studies in human retinal cells showed that incubation with 15 *μ*M curcumin increased the cytoprotective effects against H_2_O_2_ oxidative stress through reduction of reactive oxygen species (ROS) levels mediated by an increase in HO-1 expression (Figure 1) [29]. In addition, curcumin has the ability to modulate the expression of cellular regulatory proteins, including NF-*κ*B, AKT, NRF2, and growth factors, leading to an inhibition of inflammation and protection of the cells. Several studies of *in vivo* rat models have shown occurrence of direct benefits on ocular diseases from curcumin administration. Recent DR studies on Wistar albino rats with streptozotocin-induced diabetic retinopathy showed evidence of protective effects through oral administration of curcumin [30]. Similarly, other studies in rat models suggest that curcumin is effective against the development of galactose-induced cataract, naphthalene-induced cataract, selenite-induced cataract, and diabetic cataract [31–34]. In addition to this, dietary supplementation of curcumin prevented the loss of chaperone-like activity of eye lens *α*-crystallin concerning cataractogenesis caused from diabetes, thus preventing the formation of cataract in the rat lens [35]. While curcumin shows promise as a potential natural treatment, negative side effects have continuously been shown as early as 1976 [36]. These primarily include chromosomal and DNA alterations at higher doses of curcumin [36–46]. Although it has the potential for side effects at high doses, the anti-oxidative and anti-inflammatory properties of curcumin still make it a compound of choice in the treatment and prevention of AMD, DR, and cataract. 

### 3.2. Lutein and Zeaxanthin

Lutein and zeaxanthin are carotenoids that are referred to as macular pigments due to their increased presence in the human macula and retina. They are commonly found in many fruits and vegetables, such as kale, spinach, corn, kiwi, or red grapes [47]. Many epidemiologic studies on AMD, including AREDS and the Case Control Study Group for Eye Diseases of the United States, evaluated the correlation between increased blood levels of lutein and zeaxanthin and decreased risks of developing AMD [48]. While the mechanisms of action of lutein and zeaxanthin are still under investigation, it is suggested that these carotenoids may protect the macula and photoreceptor outer retinal segments from oxidative stress by triggering the antioxidant cascade that disables reactive oxygen species (Figure 1) [49]. In addition, lutein and zeaxanthin act as light filters in the eye and absorb blue-light entering the retina, hence effectively protecting the retina during acute light exposure and high light levels from LIRD [50]. Studies on cultured ARPE-19 cells showed evidence that supplementation of lutein and zeaxanthin reduced photo-oxidative damages and inhibited the expression of inflammation-related genes in RPE cells [51, 52]. In addition, results from various epidemiological studies have shown inverse associations between the amount of macular pigment and the incidence of AMD. A recent clinical study showed that supplementation with lutein and zeaxanthin improved visual function and prevented progression of the pathology in patients with early AMD [53, 54]. Similarly, clinical studies showed that zeaxanthin improves visual function in older male patients with AMD [55]. Therefore, increase of macular pigments through dietary supplements of lutein and zeaxanthin may provide a valuable option in the prevention of macular degeneration [56].

### 3.3. Saffron

Saffron is a spice frequently used in traditional medicine for its antitoxic properties. Its active ingredients, crocin and crocetin, are known antioxidant carotenoids and have antiapoptotic properties through protection of cells against reactive oxygen species [82, 83]. Crocin suppresses apoptosis, membrane lipid peroxidation, and caspase-3 activation in serum-deprived PC12 cells in hypoxic conditions. It also increases glutathione (GSH) levels and averts the activation of the JNK pathway, which contributes to the downstream signaling cascade of ceramide (Figure 1) [84]. Decreased levels of GSH lead to a higher sensitivity of the cell to apoptosis-inducing agents; thus, maintenance of the GSH levels through dietary supplementation of saffron may protect the cells from damages and death [85]. Studies on the retinal ganglion cell line RGC-5 showed that supplementation with crocin inhibited oxidative stress by decreased production of caspase-3 and -9, therefore preventing RGC-5 cell death [86]. Several studies analyzed the effects of saffron as a dietary complement in rats and in human clinical trials. In rats fed with saffron supplements, the effects of continuous bright light exposure were significantly diminished [84]. In human clinical trials of patients with early AMD, 20 mg per day saffron supplementation for 90 days showed a significant improvement of macular photopic flash electroretinogram (fERG) parameters, such as amplitude and modulation threshold [58]. While the mechanisms of the beneficial effects of saffron on the photoreceptors and bipolar cells are not yet elucidated, oral supplementation of saffron displayed a significant improvement in macular function. Preclinical studies have shown that saffron exhibits neuroprotective properties, and previous studies on rats provide evidences of cell death inhibition when exposed to intense light [84]. The results of the clinical trials indicated that dietary supplementation of saffron may induce a short-term improvement in retinal function in early AMD. In addition, the antiapoptotic and antioxidative properties of saffron have been shown to prevent formation of selenite-induced cataract in Wistar rats through inhibition of proteolysis of the lens's water-soluble protein fractions [57]. Although the studies do not conclusively prove saffron's neuroprotection in AMD and prevention of cataracts, this data seems promising in the developing of preventive and therapeutic uses of dietary supplements against the diseases. 

### 3.4. Catechin

Catechin is a polyphenolic antioxidant commonly found in green tea [87, 88]. The most abundant catechin in green tea is epigallocatechin gallate (EGCG), which has extremely strong antioxidative properties [87, 88]. Previous studies involving intraocular injection of EGCG with sodium nitroprusside showed a protective effect on the retinal photoreceptors, indicating that EGCG may benefit patients suffering from ocular diseases in which oxidative stress is involved [89]. Mechanisms of catechin's action include the destruction of oxygen free radicals, oxidative alterations of LDL, and reduction of glutamate toxicity through LPO and protein modification [90]. Studies performed on rat models involving oral administration of EGCG reduced light-induced retinal neuronal death, suggesting that EGCG may be used in preventing photoreceptor cell death [67, 91, 92]. Similarly, supplementation of catechin on N-methyl-N-nitrosourea-induced cataracts in Sprague-Dawley rats displayed inhibition of cataract-induced apoptosis in the lens epithelium, which may prove beneficial in the treatment or prevention of cataract in human patients [61]. In addition, EGCG has the ability to inhibit RPE cell migration and adhesion, thereby providing potential preventive actions against AMD [59, 60]. Therefore, catechin would be a compound of choice for prevention and treatment of diseases such as AMD. 

### 3.5. *Ginkgo biloba* Extract

*Ginkgo biloba* is one of the oldest living tree species, and its leaves have been extensively studied for their potential therapeutic properties. Ginkgo leaves contain two main active ingredients, flavonoids and terpenoids. *Ginkgo biloba* extract (GBE) is the most commonly used natural supplement in Europe and the United States, and its main properties are protection against free radical damage and lipid peroxidation. Studies suggest that GBE conserves mitochondrial metabolism and ATP production in tissues, thus partially inhibiting morphologic distortion and signs of oxidative damages due to mitochondrial aging [93–95]. Studies on mammalian cells indicate that GBE has the ability to scavenge nitric oxide and may prevent its production, consequently protecting mammalian cells against nitric-oxide reactivity [96]. Through preventing the loss of retinal ganglion cells and atrophy of the optic nerve, these properties of GBE may protect the optic nerve from degeneration, thus preventing blindness in patients suffering from glaucoma, DR, and RP [62]. Ma et al. performed studies on Sprague-Dawley rats by injecting GBE, followed by crushing the optic nerve. Animals that received the GBE extract via intraperitoneal injection prior to the optic nerve crush displayed a significantly higher survival rates of retinal ganglion cells than the controls [97]. Recent studies on Kunming mice showed that EGB761, the most widely studied GBE in clinical research [58], inhibited apoptosis of photoreceptor cells and increased cell survival after damaging or intense light exposure [98]. In addition, EGB761 was found to prevent inflammation associated with retinal detachment following the induction of vitreoretinopathy, therefore decreasing the occurrence of retinal detachment [62]. *Ginkgo biloba* is also believed to have good therapeutic potential in cases of normal tension glaucoma, where the disease continues to progress despite normalized IOP via surgery [99]. Thus, GBE could have a significant impact both for patients with glaucoma and with normal IOP. While GBE does not seem to have apparent negative side effects when used independently, evidence suggests that it can result in negative interactions when combined with some modern drugs; however, this is still under investigation [100]. Since GBE may act as a neuroprotectant and prevent damage to retinal ganglion cells, this plant extract would be an interesting component for prevention and treatment of ocular diseases such as glaucoma and other major neurodegenerative retinal pathologies. 

### 3.6. Ginseng

Ginseng is the root of *Panax ginseng* and was widely used in traditional Chinese medicine. The main active ingredients of ginseng are ginsenosides, which is a group of steroidal saponins that have the ability to target many tissues and lead to a high variety of pharmacological responses [101]. Ginsenoside saponins Rb1 and Rg3 displayed inhibition of activities that lead to the apoptotic cascades, such as glutamate-induced neurotoxicity, lipid peroxidation, and calcium influx into cells when excess glutamate is present [15]. Rb1 and Rg3 have been known to suppress TNF-*α* and provide neuroprotective effects to cultured cortical cells by inhibition of the NMDA glutamate-receptor activity (Figure 2) [102, 103]. In clinical trials on patients with glaucoma, oral administration of Korean Red Ginseng (KRG) showed considerable increases in retinal blood flow in the temporal peripapillary areas. Since swelling of blood vessels and reduction of blood flow are the important risk factors for the optic nerve damage in glaucoma; increasing the retinal blood flow may be helpful in its prevention [63]. Since ginsenosides are known to inhibit TNF-*α*, ginseng may also be important in the prevention of AMD, since inflammation is one of the major risk factors for the disease [64]. Thus, ginseng's antiapoptotic and antioxidative properties show promising benefits for patients with diseases such as AMD, glaucoma, or cataract. Significant research has been done on the beneficial effects of ginseng in diabetes as well, including blood glucose reduction, weight gain control, and increased insulin production [104–106]. Very recent studies showed that, through its anti-oxidative properties, ginseng treatment significantly reduced retinal oxidative stress in diabetic mouse models [65]. While not as extensively researched as its effects on AMD and diabetes, ginseng has also demonstrated reduction of selenite-induced cataracts in rat models. Korean researchers, Lee et al., 2010, were even able to isolate the nonsaponin component of ginseng as the particular cataract-reducing agent [66]. Due to its strong anti-oxidative properties, ginseng is a highly promising compound for further research in the treatment of AMD, DR, and even cataracts.

### 3.7. Resveratrol

After an observation of low mortality due to lack of cardiovascular pathologies in France, as compared to other countries, researchers suggested that consumption of red wine might account for protective effects for human health. Red wines contain large amounts of polyphenols, a class of compounds exhibiting various properties such as inhibition of platelet aggregation, synthesis of proinflammatory and procoagulant eicosanoids, and inhibition of endothelin synthesis, which activates vasoconstriction [107–110]. In recent studies, resveratrol has significantly extended the health and survival of mice on a high calorie diet through increased sensitivity to insulin, reduced insulin-like growth factor-1 (IGF-I) levels, increased AMP-activated protein kinase (AMPK), and peroxisome proliferator-activated receptor-gamma coactivator 1alpha (PCG-1alpha) activity [111]. In addition, many studies suggested that supplementation of resveratrol reduced diabetes-induced early vascular lesion, vascular endothelial growth factor, and oxidative stress in rat and mice models [71–73]. Thus, this property may be beneficial for patients with diabetic retinopathy through prevention of cell death. Resveratrol also showed protection against injury-induced capillary degeneration and against endoplasmic reticulum stress through the inhibition of CHOP and IRE1*α* expression [68]. Since retinal ischemia is a major factor for close-angle glaucoma and diabetic retinopathy, resveratrol could be a potential novel drug for vascular dysfunction in the retina [68, 74]. There are significant evidences that resveratrol is an effective antioxidant and has the ability to inhibit lipid peroxidation of low-density lipoproteins (LDLs), prevent the cytotoxicity of oxidized LDL, and ultimately protect cells against lipid peroxidation [112–114]. In addition, resveratrol showed evidence of induction of blood flow elevation, which could prevent damages to vessels and apoptosis of optic nerve cells in patients suffering from glaucoma [69, 70]. Resveratrol may provide neuroprotection by inducing heme-oxygenase-1 and inhibits the effects of the pro-oxidant intracellular heme present in neuronal cell cultures after strokes, thus demonstrating an innovative pathway for cellular neuroprotection [115]. Studies on rats with supplementation of resveratrol demonstrated suppression of both selenite-induced oxidative stress and cataract formation through increased glutathione and decreased malonyl dialdehyde levels in the lens [116]. These properties of resveratrol could be essential in the establishment of innovative treatments and preventive interventions in major ocular diseases such as AMD, glaucoma, cataract, and diabetic retinopathy since oxidative stress is an integral part of the pathophysiology of those diseases. 

### 3.8. Danshen (*Salvia miltiorrhiza*)

*Salvia miltiorrhiza* (SM), commonly known as Asian red sage or danshen, is composed of salvianoic acid B, which is a strong, water-soluble, polyphenolic antioxidant with anti-inflammatory properties [117, 118]. A recent study investigated the effect of injected danshen on diabetic retinopathy mice. A major injury in DR is blood capillary ischemia, characterized by a change of structure, due to the thickening of the capillary basement membrane. In such cases, oxygen radicals could not be eliminated soon enough after ischemia, leading to a destruction of the permeability membrane and edema formation due to lipid peroxidation of nerve cells [77]. Injection of danshen into the retinal hypoxia-ischemia tissues may improve the recovery of blood-oxygen transport, promote the absorption of retinal hemangioma, and therefore prevent loss of vision. In addition, danshen has the ability to scavenge free radicals and may help regulate blood sugar levels in DR patients [77]. It has also shown benefits in glaucoma, reducing the damage to retinal ganglion cells after intravenous treatment with danshen [75]. Previous clinical trials suggested that danshen may stabilize the visual field in middle to late stages of glaucoma [76]. Other studies demonstrated that danshen has the ability to inhibit TNF-*α*-induced activation of NF-*κ*B and protect against the loss of retinal ganglion cells in rabbits (Figure 2) [75]. Similar to ginseng, danshen's mechanism of neuroprotection may involve inhibition of the NMDA receptor antagonist activity [119]. Preclinical studies on danshen showed evidence of promising results for patients suffering from ocular diseases involving oxidative stress such as diabetic retinopathy, AMD, and cataract. 

### 3.9. Quercetin

Quercetin is one of the most widely studied flavonoids and is found in a variety of plant foods, including black and green teas, *Brassica* vegetables, and many types of berries [120]. Interest in flavonoids arose from the decreased incidence of cardiovascular disease and increased longevity found in populations with flavonoid-rich diets, such as the Mediterranean [121]. While there are no current FDA approved quercetin-based medications, its anti-inflammatory and anti-oxidative properties have been widely investigated [122]. Recent studies on the retinal cell line ARPE-19 demonstrated the protective effects of quercetin through inhibition of proinflammatory molecules as well as direct inhibition of the intrinsic apoptosis pathway [123]. *In vitro* studies using RF/6A rhesus choroids-retinal endothelial cells showed dose-dependent inhibition of cellular migration and tube formation, important steps of retinal angiogenesis, which is a characteristic of AMD, by treatment with quercetin [78]. Further studies on human cultured RPE cells showed similar results; quercetin treatment followed by oxidative damage dose-dependently reduced cellular damage and senescence [124]. Similarly, quercetin (and other natural flavonoids) significantly reduced reactive oxygen species' (ROS) production by ascorbate/Fe_2_^+^-induced oxidative stress in retinal cell cultures [125]. However, a study by Zhuang et al. (2011) using human umbilical vein endothelial cells (HUVECs) with oxidants found a decrease in HUVECs viability when followed by treatment with quercetin. The same study also investigated quercetin treatment effects on laser-induced choroidal neovascularization *in vivo*; contrary to the untreated controls, choroidal neovascularization size was significantly diminished by administration of quercetin [126]. However, in glaucoma rat models, quercetin was found to inhibit the expression and thus blocked the neuroprotective effects of heat shock proteins (more specifically, HSP72) [127]. Quercetin is also known to have strong anticataract properties [79] through multiple pathways; however, there is no current comprehensive knowledge of all the exact mechanisms [80]. In addition to its already mentioned anti-oxidative role, it also affects sorbitol-aldose reductase, calpain protease, glycation, and epithelial cellular signaling [80]. In fact, in 2011 Gacche and Dhole used quercetin as a standard for cataract inhibition, due to its aldose reductase inhibition, when testing for similar abilities among other flavonoids [80, 81]. While quercetin is believed to be the most abundant flavonoid in the human diet [128, 129], and although there are several potential pathways, the exact mechanism by which quercetin is processed and metabolized to affect the lens and cataract is uncertain [79]. Even though there is significant evidence linking quercetin and other flavonoids to ocular and other medical benefits, further investigation is needed to determine if this promising compound is suitable as a treatment for ocular inflammatory diseases and cataract treatment.

## 4. Conclusion

Botanical compounds have been used throughout history for the prevention and treatment of various diseases. Previously, botanical supplements had not been awarded much scientific consideration; however, in the recent years, researchers and pharmaceutical companies have raised increasing interest for active ingredients from plants and nutraceuticals. Several major eye diseases in particular, AMD, glaucoma, cataract, and other retinal pathologies, are under investigation for potential beneficial effects of botanicals. These diseases can lead to ocular damage and visual problems primarily through oxidative stress, inflammation, and ocular pressure. Similarly, the active chemical ingredients in many botanicals contain strong antioxidative, anti-inflammatory, and anti-apoptotic properties. Although this review only addresses some of the well-studied and common botanical compounds for treatment of ocular diseases, there are numerous other compounds that may help with treatment of these diseases as well. While some botanical compounds, such as curcumin or quercetin, have been the subject of several studies and clinical trials, the benefits of many compounds have not been examined as extensively. In Table 1, we have summarized the compounds that have been used for either pre-clinical or clinical trials. There are several studies that indicated the mechanistic pathways of these compounds as being effective in cellular stress shown in Figures 1 and 2; however, extensive characterization is still required to bring these compounds for therapeutic research and human clinical trials. Thus, further investigation of natural plant-derived compounds, and especially their mechanisms of action, is necessary to harness the full potential of natural compounds to be used as a non-invasive and preventative complementary and/or alternative for major eye diseases.

## Figures and Tables

**Table 1:** Clinical and preclinical trials of major botanical compounds for ocular diseases. Most of the botanical compounds investigated for eye diseases are still on the pre-clinical stage with studies focusing on cells or animal models. However, several compounds such as lutein, zeaxanthin, saffron, *Ginkgo biloba* extract, and danshen have been tested in clinical trials.

	Preclinical	Clinical
Curcumin	Diabetic retinopathy: Gupta et al. [30]	
	Cataract: Suryanarayana et al. [31], Raju et al. [32], Pandya et al. [33], Manikandan et al. [34], Kumar et al. [35]	

Lutein and zeaxanthin		AMD: Ma et al. [53, 54], Richer et al. [55], Bone et al. [56]

Saffron	Cataract: Makri et al. [57]	AMD: Falsini et al. [58]

Catechin	AMD: Alex et al. [59], Chan et al. [60]	
	Cataract: Lee et al. [61]	

*Ginkgo biloba* Extract	Diabetic retinopathy: Maclennan et al. [62]	Glaucoma: Kim et al. [63]
	Retinitis pigmentosa: Maclennan et al. [62]	

Ginseng	AMD: Cho et al. [64]	
	DR: Sen et al. [65]	
	Cataract: Lee et al. [66]	

Resveratrol	Glaucoma: Osborne [67], Li et al. [68], Kwok et al. [69], Losa [70]	
	DR: Yar et al. [71], Kim et al. [72], Hua et al. [73], Li et al. [68], Osborne et al. [74]	

Danshen	Glaucoma: Zhu and Cai [75]	Glaucoma: Wu et al. [76]
	DR: Zhang et al. [77]	

Quercetin	AMD: Chen et al. [78]	
	Cataract: Stefek and Karasu [79], Shetty et al. [80], Gacche and Dhole [81]	

AMD:  age-related macular degeneration; DR: diabetic retinopathy.
